# Sustained preventive chemotherapy for soil-transmitted helminthiases leads to reduction in prevalence and anthelminthic tablets required

**DOI:** 10.1186/s40249-019-0589-6

**Published:** 2019-10-02

**Authors:** Denise Mupfasoni, Mathieu Bangert, Alexei Mikhailov, Chiara Marocco, Antonio Montresor

**Affiliations:** Department of Control of Neglected Tropical Diseases, World Health Organization, Geneva, Switzerland

**Keywords:** Soil-transmitted helminthiases, Control; morbidity, Preventive chemotherapy, Prevalence

## Abstract

**Background:**

The goal of soil-transmitted helminthiases (STH) control programmes is to eliminate STH-associated morbidity in the target population by reducing the prevalence of moderate- and heavy-intensity infections and the overall STH infection prevalence mainly through preventive chemotherapy (PC) with either albendazole or mebendazole. Endemic countries should measure the success of their control programmes through regular epidemiological assessments. We evaluated changes in STH prevalence in countries that conducted effective PC coverage for STH to guide changes in the frequency of PC rounds and the number of tablets needed.

**Methods:**

We selected countries from World Health Organization (WHO)‘s Preventive Chemotherapy and Transmission control (PCT) databank that conducted ≥5 years of PC with effective coverage for school-age children (SAC) and extracted STH baseline and impact assessment data using the WHO Epidemiological Data Reporting Form, Ministry of Health reports and/or peer-reviewed publications. We used pooled and weighted means to plot the prevalence of infection with any STH and with each STH species at baseline and after ≥5 years of PC with effective coverage. Finally, using the WHO STH decision tree, we estimated the reduction in the number of tablets needed.

**Results:**

Fifteen countries in four WHO regions conducted annual or semi-annual rounds of PC for STH for 5 years or more and collected data before and after interventions. At baseline, the pooled prevalence was 48.9% (33.1–64.7%) for any STH, 23.2% (13.7–32.7%) for *Ascaris lumbricoides,* 21.01% (9.7–32.3%) for *Trichuris trichiura* and 18.2% (10.9–25.5%) for hookworm infections, while after ≥5 years of PC for STH, the prevalence was 14.3% (7.3–21.3%) for any STH, 6.9% (1.3–12.5%) for *A. lumbricoides*, 5.3% (1.06–9.6%) for *T. trichiura* and 8.1% (4.0–12.2%) for hookworm infections.

**Conclusions:**

Countries endemic for STH have made tremendous progress in reducing STH-associated morbidity, but very few countries have data to demonstrate that progress. In this study, the data show that nine countries should adapt their PC strategies and the frequency of PC rounds to yield a 36% reduction in drug needs. The study also highlights the importance of impact assessment surveys to adapt control strategies according to STH prevalence.

**Electronic supplementary material:**

The online version of this article (10.1186/s40249-019-0589-6) contains supplementary material, which is available to authorized users.

## Multilingual abstracts

Please see Additional file 1 for translations of the abstract into the five official working languages of the United Nations.

## Background

Soil-transmitted helminths are a group of intestinal parasites that are transmitted to humans through ingestion of infective eggs or transcutaneous penetration of larvae excreted in human faeces which contaminate the soil and water sources [1]. Soil-transmitted helminthiases (STH) are among the most common neglected tropical diseases (NTDs) in developing countries. Worldwide, it is estimated that 820 million people are infected with roundworm (*Ascaris lumbricoides*), 460 million with whipworm (*Trichuris trichiura*) and 460 million with hookworms (*Necator americanus* and *Ancylostoma duodenale*) in 102 countries [2]. In 2010, these infections contributed 3.4 million disability-adjusted life-years to the global burden of disease [1].

The World Health Organization (WHO) recommends preventive chemotherapy (PC) with albendazole or mebendazole to control STH-related morbidity alongside targeted health education and improved water and sanitation [3]. PC is the regular, coordinated administration of anthelminthic medicines to population groups at risk for STH morbidity. The target populations include preschool-age children (PSAC), school-age children (SAC), women of reproductive age (WRA) and adult groups particularly exposed to STH. The 2020 WHO goal for control of STH is to treat ≥75% of PSAC and SAC in all STH endemic countries [4]. In 2017, more than 500 million SAC (69% of total SAC in need) received PC for STH globally, with 73% of implementation units reaching 75% effective coverage [5].

The main objective of the STH control programme is to eliminate morbidity in the target population by reducing the prevalence of moderate- and heavy-intensity infections to < 2% [3]. A meta-analysis conducted by Marocco et al. showed that after five years of PC, 80% of individuals with infections of moderate and heavy intensity were cured or remained with only light-intensity infection [6].

Since 2010, WHO with the support of drug producers has donated albendazole or mebendazole for the control of STH. In 2018 alone, more than 485 million tablets were donated to endemic countries [5].

The WHO manual on STH control in SAC recommends that control programmes managers collect parasitological indicators every 3–5 years of PC with effective coverage in order to measure the effect of the intervention on the health of the population at risk [4] and, eventually, to reduce the number of PC rounds if the prevalence of STH infection has reduced to a certain level using the WHO decision tree (Additional file 2) [4].

In this study we evaluated the changes in STH prevalence from countries that conducted STH PC for ≥5 years with effective treatment coverage (that is, national treatment coverage ≥75% as defined by WHO) for SAC. These data also give an indication of expected changes in prevalence if countries conduct ≥5 years of PC, and therefore also the expected number of drugs needed by endemic countries over time.

## Methods

### National PC coverage data

We accessed WHO’s Preventive Chemotherapy and Transmission control (PCT) databank, an open web-based database on PC implementation in 102 endemic countries [7], to identify countries that had implemented effective coverage of PC (defined as ≥75% national coverage) among SAC for ≥5 years.

### Epidemiological data reporting form

The Epidemiological Data Reporting Form (EPIRF) is a standardized form designed to collect epidemiological data on all diseases targeted by PC, namely STH, lymphatic filariasis (LF), onchocerciasis and schistosomiasis. Indicators reported include: survey type, number of rounds of PC delivered prior to survey, date of survey, number of people examined, and numbers of people diagnosed positive for each STH species and for overall STH. Countries receiving donated anthelminthic medicines are invited to periodically report to WHO any changes in the epidemiological situation using the EPIRF. We extracted baseline and impact EPIRF data from endemic countries previously identified as having achieved ≥5 years of effective coverage.

### Literature search

For countries in which the EPIRF was not available we reviewed the available literature on baseline and impact assessment surveys for STH prevalence. Only surveys targeting SAC were considered. We searched official publications such as national NTD master plans and Ministry of Health survey reports as well as data published in the peer-reviewed scientific literature between 2000 and 2017. For online searches we used the words: mapping, baseline, impact assessment, soil-transmitted helminths and country names using google search and MEDLINE.

### Data analysis

Studies were eligible for inclusion if, for baseline, the data were collected before the national STH control programme was initiated and, for impact assessment, the data were collected after PC was implemented for ≥5 years with effective coverage. In addition, for all studies, SAC was the study population, the information on STH prevalence overall and/or for each STH species available. From each publication identified we extracted year, type of survey, target population, prevalence of STH and of each species, number of people examined, intensity of STH infection and diagnostic tool used. For studies where prevalence was reported by species only, we calculated the prevalence of any STH infection using the following equation [4]:

$$ \frac{\left(\boldsymbol{a}+\boldsymbol{t}+\boldsymbol{h}\right)-\left(\boldsymbol{a}\ast \boldsymbol{t}+\boldsymbol{a}\ast \boldsymbol{h}+\boldsymbol{t}\ast \boldsymbol{h}\right)+\left(\boldsymbol{a}\ast \boldsymbol{t}\ast \boldsymbol{h}\right)}{\mathbf{1.06}} $$

Where *a* = prevalence of ascariasis; *t* = prevalence of trichuriasis and *h* = prevalence of hookworm infections (all expressed as a proportion).

Data were collated, cleaned, analysed and visualized using the R (Version 3.5, R core Team, Vienna, Austria) statistical program. For data in the EPIRF form, we estimated national means using weighted cluster data for each country and for each STH species. We plotted the prevalence of any STH infection and of each species before the start of PC for STH and the prevalence after ≥5 years of PC with effective coverage using pooled and weighted means. Finally, according to the WHO STH decision tree (Additional file 2) and after ≥5 years of PC with effective coverage, countries with STH prevalence < 2% should suspend PC, < 10% should conduct PC once every 2 years, and < 20% should do PC once a year. In this study, we also estimated the decrease in drug needs for countries according to the change in STH prevalence during the ≥5 years of PC.

## Results

### Number of countries that conducted ≥5 years of PC for STH

**Fig. 1:**
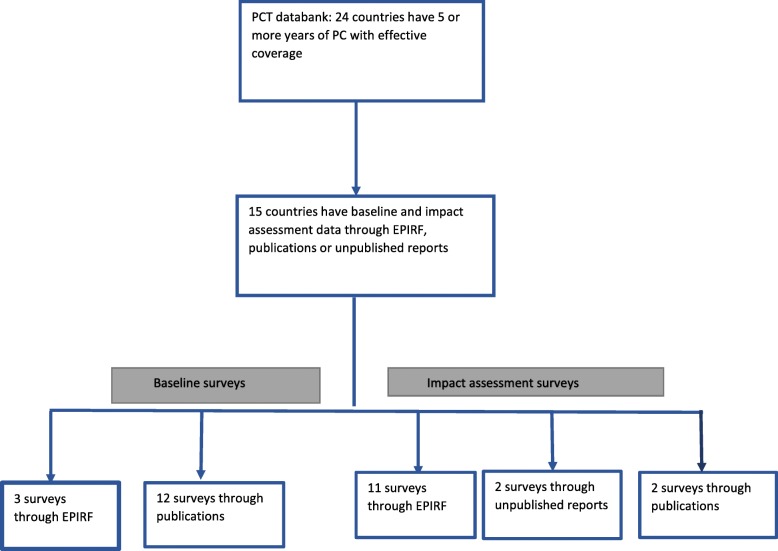
Flow chart summary on countries selected and data sources used for baseline and impact assessment surveys. SAC: School-age children; STH: Soil-transmitted helminth

From the WHO/PCT databank we identified 24 countries that conducted ≥5 years of PC for STH (Additional file 3) between 2003 and 2017. Three countries (Burkina Faso, Mali and Togo) reported their baseline data and 11 countries (Burkina Faso, Burundi, Mali, Ghana, Sierra Leone, Cameroon, Rwanda, Mexico, Nicaragua, Bangladesh and Lao People’s Democratic Republic) reported their impact assessment data through EPIRF. Two countries (Burkina Faso and Mali) collected impact assessment data for STH through transmission assessment surveys (TAS) for LF. TAS is conducted after 5 years of annual LF MDA with a coverage over 65% to confirm that the prevalence of infection is below a threshold at which recrudescence is unlikely to occur. WHO recommends collecting data on STH epidemiology simultaneously in the area to determine if STH PC should be continued after LF MDA stopped. Ten countries conducted national surveys. In addition, we identified baseline surveys conducted before PC for 12 countries and identified two impact assessments surveys through publications and two unpublished reports on STH PC impact from the Ministry of Health. In total 15 countries in four WHO regions conducted PC STH for ≥5 years and collected data before and after interventions (Fig. 1).

### Prevalence of STH infection at baseline before PC

**Fig. 2:**
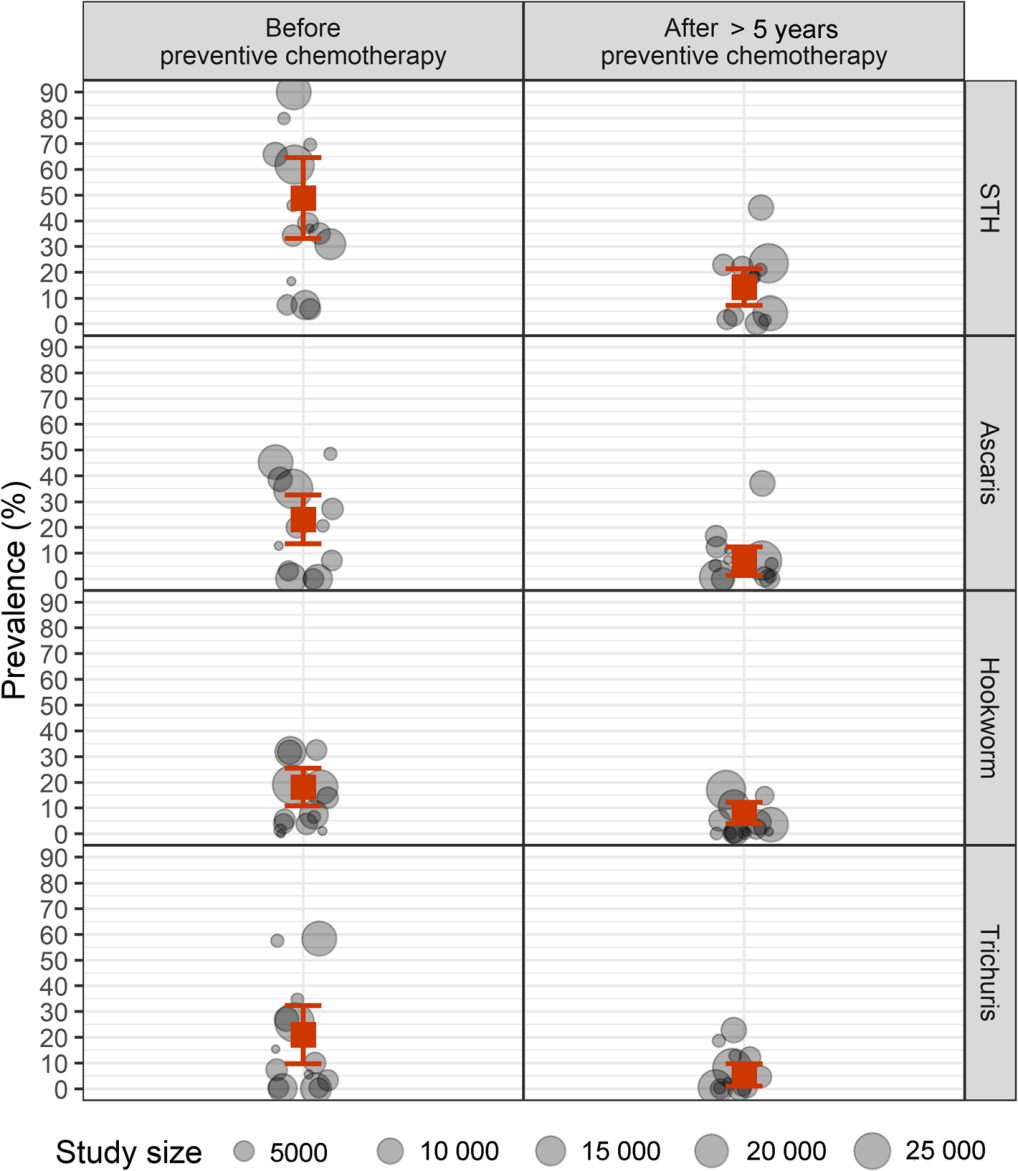
Prevalence of STH (first row), *Ascaris lumbricoides* (second row), hookworm (third row) and *Trichuris trichiura* (fourth row) infections in countries before (left column) and after (right column) ≥ 5 years of preventive chemotherapy with effective coverage. Each dot represents a published study or WHO EPIRF data. The size of the dot indicates the number of people assessed. The red square indicates pooled and weighted mean prevalence with 95% confidence intervals. WHO: World Health Organization; EPIRF: Epidemiological data reporting form

Most of the baseline data were collected during 2002–2009 (Table 1). The average size of the population surveyed was 8868 (range 266–22 166) and 80% of the surveys were designed to be nationally representative. Six, four and four countries reported high, moderate and low STH prevalence, respectively. Figure 2 (left column) shows the pooled prevalence by country; the cumulative prevalence was 48.9% (33.1–64.7%) for any STH, 23.2% (13.7–32.7%) for *A. lumbricoides,* 21.01% (9.7–32.3%) for *T. trichiura* and 18.2% (10.9–25.5%) for hookworm infections.

**Table 1:** Soil-transmitted helminth infection prevalence in SAC before and five or more years after initiation of preventive chemotherapy for STH in the 15 countries

*WHO region*	*Country*	*N*	*Ascaris lumbricoides prevalence (95 CI %)*	*Trichuris trichiura prevalence (95 CI %)*	*Hookworms prevalence (95 CI %)*	*Any STH prevalence (95% CI)*	*Year of survey [data source]*	*Diagnostic test*	*Type of survey*	*Sampled population*	Number of year of STH PC with effective coverage by 2017	Number of years between the two data collection
Africa	Burkina Faso	5146	0 (0.-0.2)	0.5 (0.3-0.7)	5.6 (3.5-8.9)	5.8 (3.7-8.9)	2004 [EPIRF]	Kato-Katz	National baseline survey	SAC	10	13
		7124	0 (0-0.1)	0 (0-0.1)	0.3 (0.1-0.6)	0.3 (0.1-0.6)	2017 [EPIRF]		Follow up through LF TAS			
	Burundi	5700	20	10	14	35	2008 [8]	Kato-Katz	National baseline survey	SAC	12	6
		12621	16.7 (14.7-18.9)	4.7 (3.9-5.6)	5.1 (4.4–5.9)	22.8 (21.2–24.4)	2014 [EPIRF]		National follow up survey			
	Mali	13318	0.1 (0-0.2)	0.3 (0.2-0.5)	7.4 (5.3-10.3)	7.4 (5.4-10.2)	2004 [EPIRF]	Kato-Katz	National baseline survey	SAC	8	10
		4672	0 (0-0)	0 (0- 0.2)	0.1 (0-0.3)	1.7 (1.2-2.5)	2014 [EPIRF]		Follow up through LF TAS			
	Ghana	4577	3.02 (2.5-3.5)	0.46 (0.26-0.66)	3.93 (3.4-4.5)	7.4	2008 [9]	Kato-Katz	National baseline survey	SAC	5	6
		7747	0.8 (0.4-1.7)	0.4 (0.2-0.8)	1.8 (1.2-2.7)	3.0 (2- 4.5)	2014 [EPIRF]		National follow up survey			
	Togo	16350	0.3 (0.2-0.5)	0.2 (0.2-0.3)	31.9 (30.4-33.5)	31 (29.6-32.4)	2009[EPIRF]	Kato-Katz	National baseline survey	SAC	5	6
		16890	NA	NA	11.1	NA	2015 [10]		National follow up survey			
	Sierra Leone	5069	7.2 (5.8-8.6)	3.3 (2.5-4.2)	32.5 (28.7-36.3)	39.1 (37.8-40.5)	2008 [11]	Kato-Katz	National baseline survey	SAC	8	7
		3632	4.4 (3-6.3)	0.8 (0.4-1.4)	14.8 (9.8-21.8)	18.2 (13.3-24.3)	2015 [EPIRF]	Kato-Katz	Not specify			
	Cameroon	22166	45.3	58.4	18	90.06	1985-1987 [12, 13]	Kato-Katz	National baseline survey	SAC	10	25
		13050	0.7 (0.3-1.8)	0.6 (0.2-1.5)	3.5 (2.7-4.4)	4.2 (3.2-5.5)	2012 [EPIRF]		National follow up survey			
	Rwanda	8312	38.6 (37.6-39.7)	27 (26.0-27.9)	31.7 (30.7-32.7)	65.8 (64.8-66.8)	2008 [14]	Kato-Katz	National baseline survey	SAC	6	6
		9251	37.2 (26.9-48.9)	22.9 (14.5-34.1)	4.5 (3.2-6.4)	45.2 (34.1-56.8)	2014 [EPIRF]		National follow up survey			
Americas	Haiti	5795	27.3	7.3	3.8	34.2	2002 [15]	Kato-Katz	National baseline survey	SAC	6	12
		3844	12.3 (11.3-13.4)	12.1 (11.1-13.2)	0.6 (0.4-0.9)	22.1 (20.8-23.4)	2014*		National follow up survey			
	Mexico	13804	20.25	15.42	NA	NA	2008 [16]	Kato-Katz	National baseline survey	SAC	8	6
		4950	11.2 (5.6-21.2)	3.7 (1.7-8.2)	0.5(0.2-1.3)	15.50(7.6-28.9)	2015 [EPIRF]		Follow up survey			
	Nicaragua	880	20.7	34.7	1.4	46	2005 [17]	Kato-Katz	cross-sectional survey	SAC	14	9
		2804	5.3 (2.3-11.7)	12.8 (7.6-20.7)	0.1(0-0.5)	18.3 (10.8-29.2)	2014 [EPIRF]		Follow up survey			
South-East Asia	Bangladesh	792	NA	NA	NA	79.8	2005 [18]	Kato-Katz	National baseline survey	SAC	6	12
		6214	7.4 (2.2-22.2)	2.7 (0.6 - 11)	0.8 (0.4-1.7)	10.9 (4.2-25.6)	2017 [EPIRF]		Follow up survey			
	Bhutan	266	12.8	5.6	1.1	16.5	2003**	Kato-Katz	National baseline survey	SAC	5	14
		1456	0.8 (0.3-1.2)	0.5 (0.2-0.9)	0.2 (0.0-0.4)	1.4 (0.8-2.0)	2017**	Mini-Flotac	National follow up survey			
	Myanmar	1000	48.5 (45.3-51.6)	57.5 (54.3-60.5)	6.5 (5.1-8.2)	69.7 (66.7-72.5)	2002 [19]	Kato-Katz	National baseline survey	SAC	12	10
		990	5.8 (4.4-7.2)	18.6 (16.2-21.0)	0.3 (0-0.6)	20.9 (18.4-23.4)	2012 [20]		National follow up survey			
Western Pacific	Lao People's Democratic Republic	29846	34.9	25.8	19.1	61.9	2002 [21]	Kato-Katz	National baseline survey	SAC	6	14
		1341	7.1 (1.8-24.5)	8.1 (1.8 - 24.5)	17.1 (5.9-40.3)	23.5 (8.2 - 51.5)	2016 [EPIRF]		Follow up survey			

*NA* Data not available, *SAC* school-aged children, *EPIRF* Epidemiological data Reporting Form, *LF* Lymphatic Filariasis, *TAS* transmission assessment survey

*Ministère de la santé publique et de la population, 2014. Evaluation de la prévalence des helminthiases intestinales chez les enfants de 6 à 15 ans scolarisés en Haïti. Unpublished

**Comprehensive school health program, Health promotion division, department of public health, Ministy of health , Royal Government of Bhutan, 2017. Report on soil-transmitted helminths survey among school children in Bhutan. Unpublished

### Prevalence of STH infection after ≥5 years of PC with effective coverage

Between 2003 and 2017, the countries included in this study conducted and reported 5–14 rounds of PC, averaging eight rounds of PC for STH for SAC with effective coverage. Eleven countries conducted national surveys to measure the impact of PC on STH infection, while two countries collected the data during LF TAS (Table 1). These surveys were conducted during 2012–2017; the average population size surveyed was 6439 (range 990–16 890). In five countries STH prevalence was moderate while in nine STH prevalence was low. The pooled prevalence was 14.3% (7.3–21.3%) for any STH, 6.9% (1.3–12.5%) for *A. lumbricoides,* 5.3% (1.06–9.6%) for *T. trichiura* and 8.1% (4.0–12.2%) for hookworm infections (Fig. 2, right column). In countries with low-level STH prevalence at the beginning of their STH control programme, the prevalence was reduced to < 2% while countries with high prevalence moved to low or moderate prevalence (Table 1).

### Estimated reduction in number of anthelminthic tablets

**Fig. 3:**
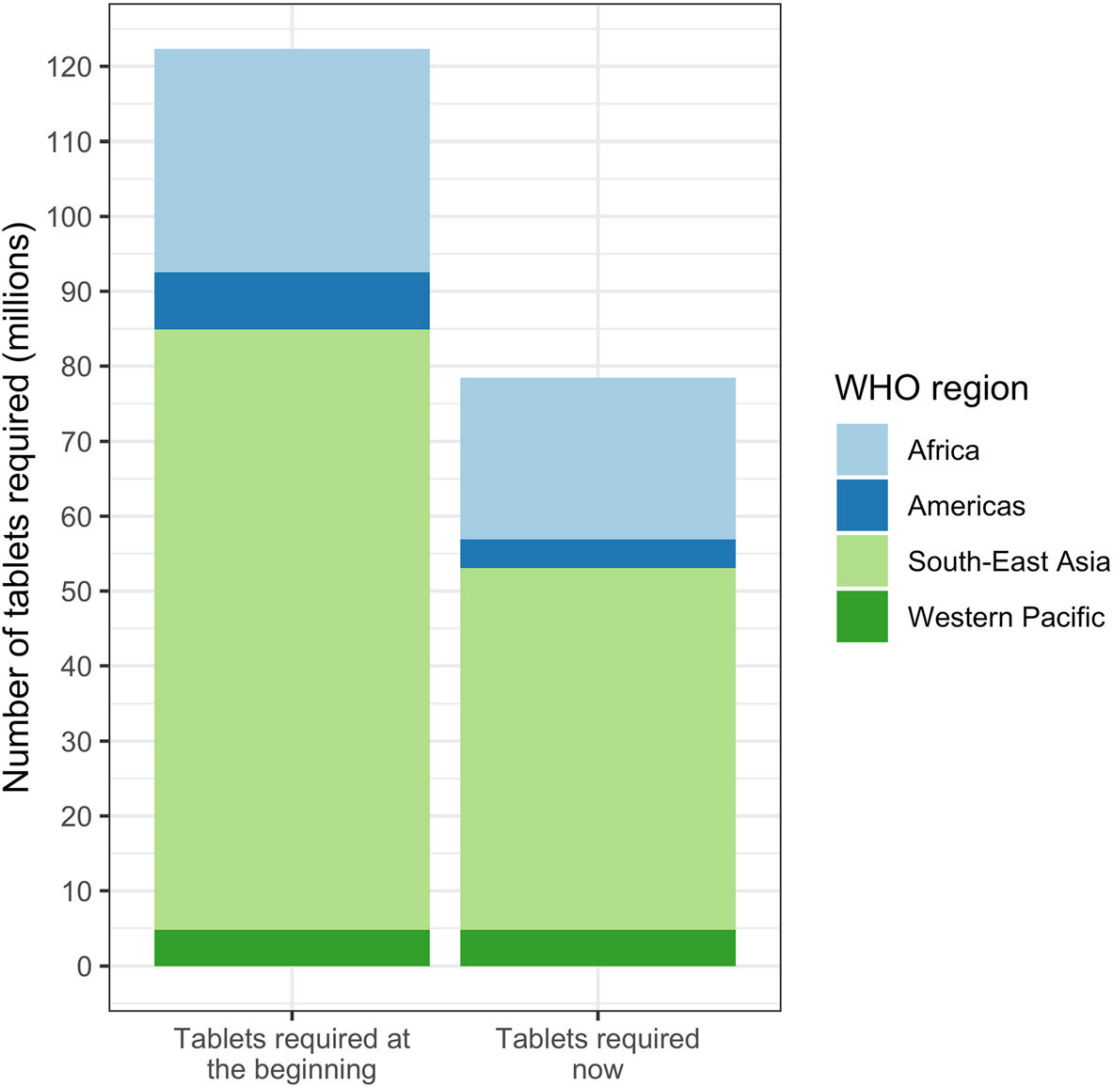
Anthelminthic drug reduction after ≥5 years of STH PC with effective coverage. STH: Soil-transmitted helminth; PC: Preventive chemotherapy

For the 5 years and more of PC for STH, five countries conducted one round annually while 10 countries did semi-annual rounds. Figure 3 shows that if the 15 countries that conducted PC for ≥5 years had applied WHO thresholds for changing the frequency of PC rounds using the impact assessment results on STH infection, the number of anthelminthic tablets needed for STH PC would have reduced by an average of 36%. According to the WHO decision tree (Additional file 2), three countries would have suspended STH PC in some areas (because the STH prevalence was < 2%) while six would have decreased the frequency of PC from semi-annual to annual or biennial, and six countries would have maintained the previous frequency of PC.

## Discussion

In this study we report data from 15 STH endemic countries that conducted ≥5 years of PC with effective coverage among SAC. The overall result demonstrates a reduction of any STH prevalence and of STH species prevalence overall, representing tremendous progress for these countries. Importantly, in three countries the prevalence of any STH in the follow up survey was less than 2%, meaning that they successfully eliminated STH morbidity.

A change in prevalence status implies a change in the frequency of PC rounds according to the WHO decision tree (Additional file 2). Countries with an STH prevalence < 2% after ≥5 years of PC should suspend the intervention and maintain surveillance to detect possible rebounds of prevalence, whereas countries with a prevalence of 2–10% should proceed to semi-annual PC. To maintain the gains made when scaling down or discontinuing PC for STH, the national STH control programme should ensure that the water, sanitation and hygiene (WASH) component is also implemented. A modelling study on the impact of deworming and WASH on STH transmission has shown that discontinuing PC and sustaining PC gains require continuation of WASH interventions [22]. Only by achieving Sustainable Development Goal 6, which seeks to ensure universal access to basic WASH in communities, schools and healthcare facilities by 2030, will reductions of morbidity due to STH infection and others NTDs associated with water and sanitation be sustained [23].

Among the 15 countries included in the study, 10 are co-endemic for LF so community-based deworming was the strategy used. Most of these countries have already started scaling down mass drug administration (MDA) for LF. Three countries (Burkina Faso, Mali and Ghana) had a low STH prevalence at the beginning of the programme, but by conducting LF MDA with ivermectin and albendazole or diethylcarbamazine citrate and albendazole, STH infections were also treated. Meanwhile, in 2019, many implementation units have already stopped PC for LF and STH.

The limitations of this study are that the data used as the baseline for STH prevalence may have been collected after the start of LF elimination programmes and thus may under-evaluate the real STH prevalence before mass treatment was initiated. For countries in which we know this was true, we used survey data collected before the start of MDA for LF, as in Cameroon, where we used baseline data collected in 1987. Moreover, impact assessment surveys were never implemented in individuals who had participated also in the baseline surveys, sometimes not even in the same area, making the comparison difficult. Finally, not all surveys were nationally representative and thus did not truly reflect the status of STH endemicity in the country. Standardized implementation of impact surveys in all endemic countries would strengthen these data.

## Conclusions

This study shows the importance of conducting impact assessment surveys after ≥5 years of STH PC with effective coverage to guide national STH control programmes in adapting the number of PC rounds according to the new epidemiological situation. Furthermore, a reduction in the frequency of PC and the consequent reduction of associated costs would help endemic countries to achieve national support for, and ownership of, their PC programmes. It would be necessary to confirm that, in different countries and in different epidemiological situation, the reduction in frequency of PC suggested by the decision tree is sufficient to maintain the improvement achieved.

## Additional files

Additional file 1: Multilingual abstracts in the five official working languages of the United Nations. (PDF 496 kb)

Additional file 2: WHO decision tree for STH control programmes. (DOCX 178 kb)

Additional file 3: Soil-transmitted helminthiases (STH) preventive chemotherapy (PC) coverage, by country, 2003–2017. (DOCX 22 kb)

## Data Availability

All relevant data are within the manuscript and its Additional files.

## Abbreviations

EPIRF: Epidemiological Data Reporting Form
LF: Lymphatic filariasis
MDA: Mass drug administration
NTD: Neglected tropical disease
PC: Preventive chemotherapy
PCT: Preventive chemotherapy and transmission control
PSAC: Preschool-age children
SAC: School-age children
STH: Soil-transmitted helminthiase
TAS: Transmission assessment surveys
WASH: Water, sanitation and hygiene
WHO: World Health Organization
