# Supplementary material for: Sustained preventive chemotherapy for soil-transmitted helminthiases leads to reduction in prevalence and anthelminthic tablets required
Source: Infect Dis Poverty. 2019 Oct 2;8:82. doi: 10.1186/s40249-019-0589-6 (PMC6774215; doi:10.1186/s40249-019-0589-6)
Supplement: Supplementary file 1 — Multilingual abstracts in the five official working languages of the United Nations. (PDF 496 kb) [file 40249_2019_589_MOESM1_ESM.pdf]

Translation of the abstract into the five official working languages of the United Nations

التخفيض في عدد الأقراص الطاردة للديدان لازم بعد خمس سنوات أو أكثر من دورات العلاج الكيميائي الناجعة بالنسبة لأدواء الديدان السارية بالتربة

دينيس موبفاسوني، ماثيو بانغارت، أليكسي ميخايلوف، كيارا ماروكو، أنتونيو مونتريسور

ملخص
الاطار: تهدف برامج مكافحة دواء الديدان السارية بالتربة (STH) الى التخلص من الاعتلالات المصاحبة لهذا الدواء في الفئة المستهدفة من خلال تقليص تفشي الاصابات المعتدلة والحادة وتقليص تفشي داء الديدان السارية بالتربة عامة باستعمال العلاج الكيميائي الوقائي (PC) سواء بالألبندازول أو بالميبيندازول. وعلى البلدان الموبوءة أن تقيس مدى نجاح برامج المكافحة من خلال التقييم الوبائي المنتظم. قيمنا التغير في نسبة انتشار دواء الديدان السارية بالتربة STH في البلدان التي أجرت علاجا كيميائيا وقائيا فعالا ضد هذا الدواء حتى نوجه التغييرات في تواتر دورات العلاج الكيميائي الوقائي PC وعدد الأقراص اللازمة.
المنهجية: انتقينا من مصرف بيانات العلاج الكيميائي الوقائي وظبط الانتقال (PCT) التابع لمنظمة الصحة العالمية WHO للبلدان التي أجرت 5 سنوات أو أكثر من العلاج الكيميائي الوقائي مع تغطية فعلية للأطفال في سن الدراسة (SAC). واستخرجنا بيانات تقييم الأثر وخط أساس أدواء الديدان السارية بالتربة STH باستعمال بلاغات البيانات الوبائية وتقارير وزارة الصحة و/أو المقالات المحكّمة. ووظفنا وجمعنا وقيمنا الوسائل الكفيلة بتقدير مدى تفشي داء الديدان السارية بالتربة STH مع كل أنواع الديدان عند نقطة الانطلاق في العلاج وبعد خمس سنوات أو أكثر من العلاج الكيميائي الوقائي ذي التغطية الفعلية. وأخيرا، بالاستناد الى مخطط تسلسل القرارات التى تعنى بدواء الديدان السارية بالتربة STH الخاص بمنظمة الصحة العالمية، قدرنا التقليص في عدد الأقراص اللازمة.
النتائج: أجرت 15 دولة من أربع أقاليم منظمة الصحة العالمية دورات سنوية أو نصف سنوية من العلاج الكيميائي الوقائي PC ضد دواء الديدان السارية بالتربة STH على امتداد خمس سنوات أو أكثر وجمعت بيانات قبل وبعد عمليات التدخل. عند خط الانطلاق، كانت نسبة التفشي المجمعة 48.9% (33.1–64.7%) في أي من داء الديدان السارية بالتربة STH: ثعبان البطن 23.2% (13.7–32.7%)، الدودة السوطية 21.01% (9.7–32.3%)، و18.2% (10.9–25.5%) للدودة الشصية.
أما بعد خمس سنوات أو أكثر من العلاج الكيميائي الوقائي PC، تقلصت نسبة التفشي الى 14.3% (7.3–21.3%) في أي من دواء الديدان السارية بالتربة STH: ثعبان البطن 6.9% (1.3–12.5%)، الدودة السوطية 5.3% (1.06–9.6%)، و8.1% (4.0–12.2%) للدودة الشصية.
الخلاصة: أحرزت البلدان الموبوءة بأدواء الديدان السارية بالتربة STH تقدما هائلا في التقليص من الاعتلالات المصاحبة لهذه الأدواء، ولكن عددا قليلا من البلدان لها بيانات لتبين هذا التقدم. تبين البيانات في هذه الدراسة أن تسعة بلدان يجب أن تكيف استراتيجيات علاجها الكيميائي الوقائي وتواتر دوراته لتحقق انخفاضا بنسبة 36% في حاجياتها من الأدوية. كما تسلط الدراسة الضوء على أهمية استطلاعات تقييم الأثر في تبني استراتيجيات المكافحة بما يتماشى مع نسبة تفشي أدواء الديدان السارية بالتربة STH.

Translated from English version into Arabic by Firas Arfaoui, Revised by Mustafa Muhammed, through

TRANSLATORS WITHOUT BORDERS

五年以上土源性蠕虫病预防性化疗后的抗蠕虫药量减少情况

Denise Mupfasoni, Mathieu Bangert, Alexei Mikhailov, Chiara Marocco and Antonio Montresor

摘要
引言：土源性蠕虫病(STH)控制项目旨在通过降低中、重度疾病的流行率来消除目标人群中 STH 相关疾病的发病率，以及通过服用阿苯达唑或甲苯达唑进行预防性化疗来降低 STH 的总体发病率。流行国家应定期通过流行病学评估来衡量其控制项目的有效性。我们在土源性蠕虫病流行国家评估了实施有效人群覆盖的预防性化疗前后的数据变化，拟用于指导其药物所需频次和数量的变化。
方法：我们从 WHO 预防性化疗和传播控制（PCT）数据库中选取了对学龄儿童（SAC）实施有效人群覆盖预防性化疗超过 5 年的国家，并从 WHO 流行病学数据报告、卫生部报告和经过同行评议的出版物中提取土源性蠕虫病基线数据和实施后效果数据。我们采用合并及加权的方法来绘制土源性蠕虫病总体及个别虫种的基线数据及实施 5 年有效人群覆盖预防性化疗后的感染率情况。最后使用 WHO 土源性蠕虫病决策树，预计了所需药片的减少数量。
结果：WHO 四个区域内开展了为期 5 或以上的每年或每半年一次预防性化疗国家有 15 个，收集干预前后的数据。在基线调查时，总体患病率为 48.9%(33.1﹣64.7%)，蛔虫为 23.2%(13.7–32.7%)，鞭虫为 21.01% (9.7–32.3%)，钩虫为 18.2%(10.9–25.5%)。而使用预防性化疗 5 年后，总体患病率为 14.3%(7.3–21.3%)，蛔虫为 6.9% (1.3–12.5%)，鞭虫为 5.3% (1.06–9.6%)，钩虫为 8.1%(4.0–12.2%)。
结论：流行区国家在降低土源性蠕虫病相关疾病的发病率方面取得了巨大进展，但很少有国家通过数据表明这一进展。本研究数据显示，9 个国家应调整其化疗战略和服药频率，使药物用量减少 36%。本研究亦强调了效果评估调查的重要性，应根据土源性蠕虫病的流行情况来调整控制策略。

Translated from English version into Chinese by Peng Song, edited by Jin Chen

<Réduction du nombre de comprimés anthelminthiques nécessairesaprès au moins cinq ans de cycles de chimiothérapie préventive efficace contre les helminthiases transmises par le sol

Denise Mupfasoni, Mathieu Bangert, Alexei Mikhailov, Chiara Maroc et Antonio Montresor

Résumé
Contexte: L'objectif des programmes de lutte contre les helminthiases transmises par le sol (HTS) est de réduire la morbidité associée à cette maladie dans la population cible en réduisant la prévalence des infections d'intensité modérée et élevée ainsi que la prévalence globale de l'infection principalement par la chimiothérapie préventive (CP) avec soit de l'albendazole ou du mebendazole. Les pays d'endémie doivent mesurer le succès de leurs programmes de lutte par le biais d'évaluations épidémiologiques régulières. Nous avons évalué les changements dans la prévalence des STH dans les pays ayant mis en place une couverture efficace avec CP pour les STH afin de guider les changements dans la fréquence des cycles de CP et le nombre de comprimés nécessaires.
Méthodes: Nous avons sélectionné des pays de la banque de données de Chimiothérapie Préventive et de contrôle de la Transmission (CPT) de l'OMS ayant effectué une CP ≥ 5 ans avec

une couverture efficace pour les enfants d'âge scolaire (EAS) et extrait les données de base et d'évaluation de l'impact sur la STH à l'aide du formulaire de déclaration des données épidémiologiques de l'OMS, Rapports du Ministère de la Santé et/ou publications examinées par des pairs. Nous avons utilisé des moyens regroupés et pondérés pour tracer la prévalence de l'infection avec n'importe quelle HTS et avec chaque espèce d'HTS au départ et après ≥ 5 ans de CP avec une couverture efficace. Enfin, en utilisant l'arbre de décision de l'OMS pour les HTS, nous avons estimé la réduction du nombre de comprimés nécessaires.

Résultats: Quinze pays dans quatre régions de l'OMS ont organisé des cycles annuels ou semestriels de CP pour les HTS pendant 5 ans ou plus et ont collecté des données avant et après les interventions. Au départ, la prévalence regroupée était de 48,9% (33,1 à 64,7%) pour toute HTS, de 23,2% (13,7 à 32,7%) pour *Acaris lumbricoides*, de 21,01% (9,7 à 32,3%) pour *Trichuris trichiura* et de 18,2% (10,9 à 25,5%).) pour les infections par les ankylostomes, alors qu'après ≥ 5 ans de CP pour les HTS, la prévalence était de 14,3% (7,3 à 21,3%) pour les HTS, 6,9% (1,3 à 12,5%) pour *A. lumbricoides*, 5,3% (1,06 à 9,6%) pour *T. trichiura* et 8,1% (4,0–12,2%) pour les infections par les ankylostomes.

Conclusions: Les pays d'endémie pour l'HTS ont réalisé des progrès considérables dans la réduction de la morbidité associée à l'HTS, mais très peu de pays disposent de données pour démontrer ces progrès. Dans cette étude, les données montrent que neuf pays devraient adapter leurs stratégies en matière de CP et la fréquence des cycles de CP afin de réduire de 36% les besoins en médicaments. L'étude souligne également l'importance des enquêtes d'évaluation de l'impact pour adapter les stratégies de contrôle en fonction de la prévalence des HTS.

Translated from English version into French by Sarah Merlin, Revised by Imane Bouamoud, through

TRANSLATORS WITHOUT BORDERS

Необходимость в сокращении числа антигельминтных таблеток после пяти или более лет эффективных циклов профилактической химиотерапии при гельминтозах, передающихся через почву

Денисе Мупфасони, Матье Бангер, Алексей Михайлов, Киара Марокко и Антонио Монтресор

Аннотация

Предпосылки: Целью программ борьбы с гельминтозами, передаваемыми через почву (ГПП), является устранение связанной с ГПП заболеваемости в целевой популяции путем снижения распространенности инфекций средней и тяжелой степени и общей распространенности инфекции ГПП в основном путем профилактической химиотерапии (ПХ) с применением альбендазола или мебендазола. Эндемичные страны должны оценивать успех своих программ борьбы с помощью регулярных эпидемиологических оценок. Мы

оценили изменения в распространенности ГПП в странах, где проводилось эффективное использование ПХ при ГПП, чтобы определить изменения в частоте циклов ПХ и количестве необходимых таблеток.

Методы: Мы выбрали те страны из банка данных ВОЗ по профилактической химиотерапии и контролю передачи (ПХП), в которых была проведена ≥ 5 лет ПХ с эффективным охватом детей школьного возраста (ДШВ) и извлекли базовые данные по ГПП и данные оценки воздействия с использованием формы отчетности по эпидемиологическим данным ВОЗ, отчетам Министерства здравоохранения и/или рецензируемым публикациям. Мы использовали объединенные и взвешенные средние значения для определения распространенности инфекции с любым ГПП и с каждым видом ГПП в начале исследования и после ≥ 5 лет ПХ с эффективным охватом. Наконец, используя схему принятия решений ВОЗ по ГПП, мы определили необходимое сокращение количества таблеток.

Результаты: Пятнадцать стран в четырех регионах ВОЗ провели ежегодные или полугодовые циклы ПХ при ГПП в течение 5 или более лет и собирали данные до и после вмешательства. В начале исследования общая распространенность составляла 48,9% (33,1–64,7%) для любого ГПП, 23,2% (13,7–32,7%) для *аскарид,* 21,01% (9,7–32,3%) для *власоглавов* и 18,2% (10,9–25,5%) для нематодных инфекций, в то время как после ≥ 5 лет ПХ для ГПП распространенность составила 14,3% (7,3–21,3%) для любой СТГ 6,9% (1,3–12,5%) для *аскарид*, 5,3% (1,06–9,6%) для *власоглавов* и 8,1% (4,0–12,2%) для нематодных инфекций.

Выводы: Страны, эндемичные по ГПП, достигли огромного прогресса в снижении смертности, связанной с ГПП, но лишь немногие страны имеют данные, демонстрирующие такой прогресс. В этом исследовании на данных показано, что девять стран должны адаптировать свои стратегии ПХ и частоту циклов ПХ, чтобы добиться снижения потребности в лекарствах на 36%. В исследовании также подчеркивается важность исследований по оценке воздействия для адаптации стратегий контроля в соответствии с распространенностью ГПП.

Translated from English version into Russian by Anna Kukharchuk, Revised by Alexander Somin, through

TRANSLATORS
WITHOUT BORDERS

Reducción de la cantidad de tabletas necesarias de antihelmínticos luego de cinco o más años de rondas efectivas de quimioterapia preventiva para las helmintiasis transmitidas por el suelo

Denise Mupfasoni, Mathieu Bangert, Alexei Mikhailov, Chiara Marocco y Antonio Montresor

Resumen

Antecedentes: El objetivo de los programas de control de las helmintiasis transmitidas por el suelo (HTS) es eliminar la morbilidad asociada a las HTS en la población blanco mediante la reducción

de la prevalencia de infecciones de intensidad moderada o alta y de la prevalencia global de HTS, principalmente a través de la quimioterapia preventiva (QP) con albendazol o mebendazol. Los países en donde la enfermedad es endémica deben medir el éxito de sus programas de control a través de evaluaciones epidemiológicas regulares. Evaluamos los cambios en la prevalencia de HTS en los países que llevaron a cabo una cobertura efectiva con QP para orientar los cambios en la frecuencia de las rondas de QP y en la cantidad de tabletas necesarias.

Métodos: Seleccionamos los países del banco de datos de Quimioterapia Preventiva y Control de la Transmisión (QPT) de la OMS que realizaron ≥ 5 años de QP con cobertura efectiva para los niños en edad escolar. Extrajimos datos basales acerca de las HTS y datos de la evaluación del impacto utilizando el formulario para reporte de datos epidemiológicos de la OMS, informes del Ministerio de Salud o publicaciones revisadas por expertos. Utilizamos las medias para datos agrupados y ponderados para determinar la prevalencia de infección con cualquiera y con cada una de las especies de HTS al inicio y tras ≥ 5 años de cobertura efectiva con QP. Finalmente, utilizamos el diagrama de decisiones para las HTS de la OMS para estimar la reducción en la cantidad de tabletas necesarias.

Resultados: Quince países de cuatro regiones de la OMS llevaron a cabo rondas de tratamiento de QP anuales o bianuales para HTS durante 5 años o más y recopilaron información antes y después de las intervenciones. Al inicio, la prevalencia agrupada fue del 48,9 % (33,1–64,7 %) para cualquier HTS, 23,2 % (13,7–32,7 %) para *Ascaris lumbricoides*, 21,01 % (9,7–32,3 %) para *Trichuris trichiura* y 18,2 % (10,9–25,5 %) para infecciones por anquilostomas; mientras que después de ≥5 años de QP para HTS, la prevalencia fue del 14,3 % (7,3–21,3 %) para cualquier HTS, 6,9 % (1,3–12,5 %) para *A. lumbricoides*, 5,3 % (1,06–9,6 %) para *T. trichiura* y 8,1 % (4,0–12,2 %) para infecciones por anquilostomas.

Conclusiones: Los países con HTS endémica han tenido enormes progresos en la reducción de la morbilidad asociada a HTS, pero solo muy pocos países tienen información que lo demuestre. En este estudio, los datos muestran que nueve países deberían adecuar sus estrategias de QP y la frecuencia de las rondas de tratamiento para lograr una reducción del 36 % en la necesidad de fármacos. El estudio también subraya la importancia de la evaluación del impacto para adecuar las estrategias de control de acuerdo a la prevalencia de HTS.

Translated from English version into Spanish by Gabriel Cano, Revised by Maria Patricia Arce, through

TRANSLATORS
WITHOUT BORDERS
