# Supplementary figures and images for: Sustained preventive chemotherapy for soil-transmitted helminthiases leads to reduction in prevalence and anthelminthic tablets required
Source: Infect Dis Poverty. 2019 Oct 2;8:82. doi: 10.1186/s40249-019-0589-6 (PMC6774215; doi:10.1186/s40249-019-0589-6)

**Additional file 1. WHO decision tree for STH control programmes**


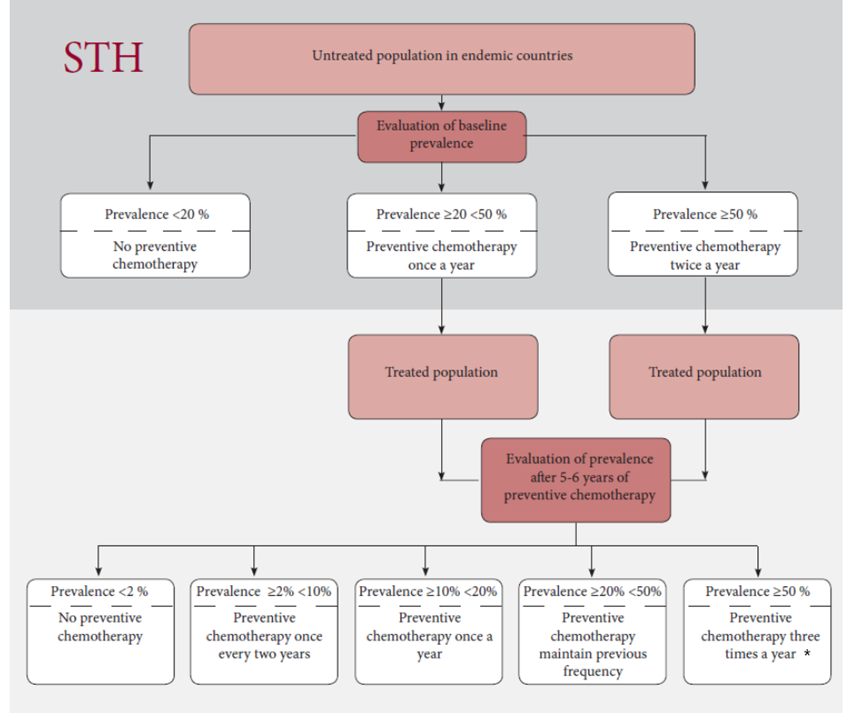

Supplement: Supplementary file 2 — WHO decision tree for STH control programmes. (DOCX 178 kb) [file 40249_2019_589_MOESM2_ESM.docx]
