# Supplementary material for: Sustained preventive chemotherapy for soil-transmitted helminthiases leads to reduction in prevalence and anthelminthic tablets required
Source: Infect Dis Poverty. 2019 Oct 2;8:82. doi: 10.1186/s40249-019-0589-6 (PMC6774215; doi:10.1186/s40249-019-0589-6)
Supplement: Supplementary file 3 — Soil-transmitted helminthiases (STH) preventive chemotherapy (PC) coverage, by country, 2003–2017. (DOCX 22 kb) [file 40249_2019_589_MOESM3_ESM.docx]

Additional file 2. Soil-transmitted helminthiases (STH) preventive chemotherapy (PC) coverage, by country, 2003–2017

|  |  | **PC coverage (%)** | | | | | | | | | | | | | |  |  |  |
| --- | --- | --- | --- | --- | --- | --- | --- | --- | --- | --- | --- | --- | --- | --- | --- | --- | --- | --- |
| **WHO region** | **Country** | **2003** | **2004** | **2005** | **2006** | **2007** | **2008** | **2009** | **2010** | **2011** | **2012** | **2013** | **2014** | **2015** | **2016** | **2017** | **No. of rounds** | **No. of effective rounds** |
| African | Burkina Faso | 44.30 | 77.60 | 77.64 | 80.18 | 81.53 | 82.27 | 81.70 | 1.96 | 94.64 | 95.56 | 81.68 | 53.99 | 55.46 | 100.00 | 0.00 | 14 | 10 |
| African | Burundi | No data | 87.68 | 100.00 | 10.12 | 74.79 | 84.58 | 100.00 | 100.00 | 100.00 | 100.00 | 100.00 | 100.00 | 100.00 | 97.32 | 99.99 | 13 | 12 |
| African | Cameroon | No data | No data | No data | 3.32 | 82.87 | 88.19 | 65.28 | 85.82 | 100.00 | 100.00 | 77.26 | 100.00 | 100.00 | 90.60 | 78.39 | 11 | 10 |
| African | Ghana | 12.52 | 21.86 | 26.83 | 39.92 | 26.24 | 49.72 | 100.00 | 100.00 | 100.00 | 100.00 | 100.00 | 49.72 | 37.92 | 59.00 | 16.14 | 14 | 5 |
| African | Malawi | 4.44 | 10.32 | 6.62 | 12.55 | No data | 21.05 | 70.98 | 100.00 | 84.00 | 100.00 | 92.04 | No data | 100.00 | 91.50 | 74.96 | 12 | 6 |
| African | Mali | 6.64 | 4.86 | 81.85 | 60.61 | 39.23 | 43.98 | 80.93 | 100.00 | 100.00 | 78.26 | 98.79 | 13.35 | 98.30 | 100.00 | 0.00 | 14 | 8 |
| African | Rwanda | No data | No data | No data | No data | No data | 99.94 | 100.00 | 100.00 | 100.00 | 92.56 | 91.81 | 100.00 | 98.58 | 100.00 | 99.08 | 9 | 10 |
| African | Sierra Leone | 11.92 | 19.36 | 31.62 | 62.87 | 44.89 | 54.50 | 94.07 | 100.00 | 100.00 | 100.00 | 100.00 | No data | 100.00 | 95.81 | 77.98 | 13 | 8 |
| African | Togo | 15.36 | 15.68 | 16.04 | 16.20 | 16.10 | 15.31 | 5.29 | 13.29 | 60.50 | 87.44 | 82.44 | 74.01 | 100.00 | 99.79 | 99.96 | 14 | 5 |
| Americas | Belize | No data | No data | 6.72 | 55.09 | 42.07 | 29.78 | 100.00 | 100.00 | 100.00 | 100.00 | 100.00 | No data | No data | 100.00 | 0.00 | 10 | 6 |
| Americas | Dominican Republic | 2.67 | 4.24 | 2.85 | 10.89 | 92.20 | 77.45 | 100.00 | 100.00 | 100.00 | 100.00 | 100.00 | 100.00 | 100.00 | 100.00 | 80.25 | 14 | 11 |
| Americas | Haiti | 12.73 | 35.29 | 44.43 | 26.84 | 100.00 | 40.97 | 89.88 | 41.59 | 89.42 | 100.00 | 73.86 | 91.35 | 92.64 | 72.90 | 24.16 | 14 | 6 |
| Americas | Mexico | 55.07 | 59.73 | 59.14 | 59.44 | No data | No data | 100.00 | 100.00 | 100.00 | 100.00 | 100.00 | 100.00 | 100.00 | 100.00 | 62.84 | 12 | 8 |
| Americas | Nicaragua | 71.57 | 78.79 | 86.28 | 100.00 | 91.29 | 88.76 | 100.00 | 86.79 | 75.88 | 80.35 | 100.00 | 100.00 | 100.00 | 100.00 | 96.82 | 14 | 14 |
| European | Kyrgyzstan | No data | No data | No data | No data | No data | No data | 100.00 | 100.00 | 100.00 | No data | No data | 100.00 | 100.00 | No data | 100.00 | 5 | 6 |
| European | Tajikistan | No data | No data | No data | No data | 2.19 | 2.20 | No data | No data | No data | 100.00 | 100.00 | 100.00 | 100.00 | 100.00 | 99.88 | 7 | 6 |
| South-East Asia | Bangladesh | 4.19 | 11.15 | 12.00 | 12.47 | 16.99 | 23.47 | 35.93 | 34.62 | 26.59 | 88.50 | 85.88 | 88.56 | 86.40 | 86.37 | 95.36 | 14 | 6 |
| South-East Asia | Bhutan | 81.81 | 73.33 | 76.15 | 81.69 | No data | No data | No data | No data | No data | No data | No data | No data | 100.00 | 98.52 | 0.00 | 6 | 5 |
| South-East Asia | Democratic People's Republic of Korea | 0.36 | No data | No data | No data | 91.82 | 85.24 | 100.00 | 100.00 | 100.00 | 100.00 | 100.00 | 100.00 | 98.69 | 99.39 | 67.44 | 11 | 10 |
| South-East Asia | Myanmar | 16.35 | 43.70 | 18.03 | 77.89 | 85.90 | 87.49 | 85.98 | 94.64 | 100.00 | 100.00 | 87.00 | 95.35 | 99.18 | 97.49 | 97.83 | 14 | 12 |
| Western Pacific | Cambodia | 60.40 | 64.79 | 73.75 | 81.61 | 81.43 | 79.75 | 74.34 | 83.34 | 76.48 | 87.61 | 91.79 | 95.07 | 95.99 | 95.29 | 91.82 | 14 | 11 |
| Western Pacific | Kiribati | 40.63 | 73.15 | 79.45 | No data | No data | No data | No data | 100.00 | 100.00 | 100.00 | 100.00 | 56.54 | 69.01 | 74.41 | 0.00 | 10 | 5 |
| Western Pacific | Lao People's Democratic Republic | 9.76 | 9.47 | 19.53 | 61.61 | 61.74 | 93.94 | 58.65 | 61.38 | 58.10 | 61.39 | 88.61 | 89.72 | 91.60 | 91.45 | 94.63 | 14 | 6 |
| Western Pacific | Tuvalu | 81.27 | 82.97 | 80.63 | No data | No data | No data | No data | 68.85 | 87.57 | 84.16 | 86.94 | 100.00 | 76.17 | 72.46 | 0.00 | 10 | 8 |
| Data source: WHO PC databank | |  |  |  |  |  |  |  |  |  |  |  |  |  |  |  |  |  |
